# Re-description of the loach species *Leptobotia citrauratea* (Teleostei, Botiidae), with the description of *L. brachycephala* from southern Zhejiang Province, China

**DOI:** 10.3897/zookeys.1017.57503

**Published:** 2021-02-12

**Authors:** Dong-Ming Guo, E Zhang

**Affiliations:** 1 Institute of Hydrobiology, Chinese Academy of Sciences, Wuhan, 430072, Hubei Province, China Institute of Hydrobiology, Chinese Academy of Sciences Wuhan China; 2 University of Chinese Academy of Sciences, Beijing, 100049, China University of Chinese Academy of Sciences Beijing China

**Keywords:** Biodiversity, Cypriniformes, morphology, phylogeny, taxonomy

## Abstract

*Leptobotia citrauratea* (Nichols, 1925), a loach species, originally described from Dongting Lake, was recently rehabilitated, based on the examination of the holotype and non-topotypical specimens. Several field surveys conducted from 2016 to 2019 in Zhejiang, Jiangxi and Hunan Provinces, P.R. China, yielded many specimens of *Leptobotia* which were initially identified as *L. citrauratea*. Molecular and morphological analyses of these specimens demonstrated that two distinct species are involved. One was identified as *L. citrauratea*, represented by specimens from both the Poyang and Dongting Lake (type locality) systems in Jiangxi and Hunan Provinces, and the other species is described as *L. brachycephala*, represented by specimens from the Ou-Jiang and Qu-Jiang, two coastal rivers of Zhejiang Province, China. *Leptobotia brachycephala* resembles *L. citrauratea* and *L. micra* in having a row of orange dots or an orange stripe along the dorsal mid-line of the body, extending from the nape to the caudal-fin base – a unique character in *Leptobotia*. *Leptobotia brachycephala* differs from *L. citrauratea* and *L. micra* Bohlen & Šlechtová, 2017, in caudal-fin shape and pelvic-fin insertion and proportional measurements including caudal-fin length, head length, predorsal length and anal-fin length. Its species status was further corroborated by position in a molecular phylogenetic analysis, based on the mitochondrial cyt b gene and its minimum uncorrected p-distance (2.9%) from congeneric species.

## Introduction

The loach genus *Leptobotia* was erected by Bleeker (1870) with the simultaneously-described *Leptobotia elongata* (Bleeker, 1870) as type species by monotypy. The genus is distinguished from other genera of the family Botiidae by the presence of a simple suborbital spine beneath the eye (Tang et al. 2008). Sixteen species are currently included in *Leptobotia* (Kottelat 2012; Bohlen and Šlechtová 2016, 2017). The majority of these species are known from southern China, mainly in the Yangtze River (= Chang-Jiang) and Pearl River (= Zhu-Jiang) Basins and coastal rivers of southern Zhejiang Province. Two species – *L. flavolineata* Wang, 1981 and *L. orientalis* Xu, Fang & Wang, 1981 – occur in northern China (Tang et al. 2008; Kottelat 2012; Bohlen and Šlechtová 2017).

Nichols et al. (1925) described *Leptobotia citrauratea* from the Dongting Lake system in Hunan Province, China. Chen (1980) considered specimens of *L. citrauratea* to be juveniles of *L. elongata*. The synonymy of *L. citrauratea* with *L. elongata* was generally accepted by subsequent researchers (Kottelat 2004, 2012). However, Nalbant (2002) regarded *L. citrauratea* to be a valid species. Bohlen and Šlechtová (2017), based on examination of the holotype and non-topotypical specimens from the Poyang Lake system in Jiangxi Province, southern China, recognised *L. citrauratea* as a valid species. A row of orange dots or an orange stripe along the dorsal mid-line distinguishes it from *L. elongata*.

Several field surveys conducted by us from 2016 to 2019 in Zhejiang, Jiangxi and Hunan Provinces, yielded many specimens of *Leptobotia* with a row of orange dots or an orange stripe along the dorsal mid-line and orange or yellowish-brown lateral portion, by which they were initially identified as *L. citrauratea*. These specimens were recovered in two distinct lineages in a phylogenetic analysis, based on the mitochondrial cytochrome *b* (cyt b) gene sequences. Morphological analysis also indicated that two distinct species are involved. One of them was identified as *L. citrauratea*, represented by specimens sampled from the Poyang and Dongting Lake systems. The other species is an undescribed species represented by specimens from the Ou-Jiang and Qu-Jiang in Zhejiang Province. The present study aims to provide a re-description of *L. citrauratea*, based on fresh topotypical specimens and the formal description of the undescribed species.

## Materials and methods

Specimens were either initially fixed in 10% formalin and then transferred to 70% ethanol for morphological examination or preserved in 95% ethanol for DNA extraction. Seventy-three specimens from the three species (*L. citrauratea*, *L. elongata* and *L. brachycephala*) were used for morphometric analysis. Voucher specimens are kept in the ichthyological collection of the Museum of Aquatic Organisms at the Institute of Hydrobiology (**IHB**), Chinese Academy of Sciences, Wuhan City, Hubei Province, China.

Twenty-five measurements (Tables 1, 2) were taken from 22 specimens of *Leptobotia elongata* collected from the upper Chang-Jiang Basin in Sichuan Province; 29 specimens of *L. citrauratea* from the Gan-Jiang (an effluent of Poyang Lake) and Dongting Lake; and 22 specimens of *L. brachycephala* from the Ou-Jiang and Qu-Jiang. Measurements were taken point to point with digital calipers directly linked to a data-recording computer and data recorded to the nearest 0.1 mm. All measurements and counts were made on the left side of each specimen, following the methods of Kottelat (2001) and Xin et al. (2009). The lateral head length and measurements of other parts of the body were given as percentages of the standard length (SL) and measurements of parts of the head were expressed as proportions of the lateral head length (HL). Morphometric variation was analysed with Principal Component Analysis (PCA) in Past v.1.89 (Hammer et al. 2009). The PCA was made with log-transformed measurement data to a tenth of a millimetre in a covariance matrix and without rotation.

**Table 1.** Morphometric measurements for three species of *Leptobotia*: *L. citrauratea*, *L. elongata* and *L. brachycephala*.

	*L. citrauratea*					*L. elongata*		*L. brachycephala* sp. nov.		
	Dongting Lake (n = 17)			Poyang Lake (n = 12)		Chang-Jiang (n = 22)		Holotype	Paratypes (n = 21)	
	Range Mean±SD			Range Mean±SD		Range Mean±SD			Range	Mean±SD
SL (mm)	47.0–65.3		52.9±4.2	33.3–42.7	37.5±2.6	97.8–272.0	180.2±52.1	63.9	43.7–66.8	56.1±5.7
**Morphometric data**										
% **of SL**										
Body depth	14.8–18.8		16.7±1.4	15.7–19.2	17.2±1.0	17.3–23.7	20.0±1.7	12.9	11.1–15.8	12.8±1.1
Body width at dorsal origin	8.7–12.5		10.5±1.1	9.7–12.4	11.4±0.8	8.3–14.9	11.4±1.5	7.6	6.3–9.7	8.3±0.9
Head length	22.5–25.6		24.2±0.8	23.8–26.8	25.4±0.8	24.4–31.4	27.8±1.8	19.9	18.4–22.8	20.6±1.1
Dorsal-fin length	14.9–18.0		16.9±0.9	13.7–16.8	14.9±1.0	16.3–22.0	18.7±1.6	10.6	9.0–11.6	10.3±0.7
Pectoral-fin length	14.8–21.1		17.0±2.0	13.8–17.5	15.6±1.0	14.4–18.2	16.8±0.9	12.8	9.6–14.2	11.9±1.1
Pelvic-fin length	12.7–16.9		13.7±1.1	12.1–14.3	13.3±0.6	13.3–16.3	14.9±0.7	10.2	9.1–12.6	10.6±0.9
Anal-fin length	14.7–17.7		16.3±0.8	13.0–15.9	14.2±0.9	15.8–20.2	18.0±1.2	9.3	8.1–11.4	10.0±0.8
Upper caudal-lobe length	23.6–26.9		25.5±0.9	23.6–26.7	25.1±1.0	23.1–30.0	25.9±2.1	18.4	15.2–20.8	17.9±1.5
Median caudal-ray length	11.5–13.7		12.4±0.6	13.2–15.0	14.2±0.5	9.6–13.1	10.7±1.0	13.0	11.5–15.0	13.0±0.9
Caudal-peduncle length	12.1–15.8		13.9±0.9	12.5–14.2	13.3±0.5	13.8–17.1	15.5±0.9	17.3	14.6–20.0	17.7±1.6
Caudal-peduncle depth	10.4–12.9		11.6±0.7	11.3–13.5	12.3±0.7	10.9–13.8	12.1±0.7	11.5	10.3–14.4	11.7±1.1
Caudal-peduncle width	2.1–3.9		2.9±0.7	3.2–4.2	3.7±0.3	2.6–4.8	3.6±0.6	2.8	1.7–4.3	3.1±0.8
Predorsal length	51.4–56.3		54.0±1.2	52.8–55.3	53.9±0.7	54.5–58.8	56.5±1.2	51.7	49.9–54.7	52.7±1.6
Prepectoral length	21.7–26.8		23.7±1.1	23.4–25.5	24.5±0.6	25.1–31.6	28.3±1.8	19.7	18.9–23.4	20.5±1.1
Prepelvic length	53.2–59.0		56.5±1.7	52.3–56.0	54.4±1.2	56.0–61.5	57.8±1.6	49.2	47.9–53.1	50.9±1.5
Preanal length	76.7–81.4		79.0±1.3	75.0–81.3	78.1±1.6	75.9–79.8	77.4±1.1	74.6	70.2–76.9	73.8±1.6
Vent to anal distance	6.8–10.3		8.9±0.9	6.7–8.3	7.5±0.6	6.1–10.1	8.4±1.1	10.4	7.4–10.7	9.3±0.9
Pelvic to anal distance	19.9–25.7		22.9±1.8	20.2–23.6	21.4±1.1	16.5–23.0	19.3±1.5	24.4	20.3–24.6	22.0±1.3
% **of HL**										
Head depth at nape	56.4–63.1	59.3±1.6		55.0–62.8	58.8±2.0	53.6–64.2	57.4±2.8	51.8	49.0–56.8	52.8±2.6
Head depth at eye	42.3–48.4	45.8±2.1		43.6–49.7	47.0±1.8	40.5–48.5	43.7±2.1	38.6	37.7–45.8	41.8±2.7
Snout length	35.4–43.9	39.6±2.3		35.1–42.5	38.4±2.0	35.7–42.6	38.4±2.1	36.2	35.6–41.8	38.2±2.3
Postorbital head length	48.5–54.8	51.6–2.0		50.8–55.2	52.5±1.6	54.5–61.9	57.8±1.9	55.2	52.4–60.0	56.2–2.6
Eye diameter	9.2–12.1	10.3±1.0		9.3–10.8	10.0±0.5	4.2–8.4	6.0±1.1	11.4	9.1–12.2	10.4±0.9
Interorbital width	14.5–22.8	17.3±2.4		16.1–19.6	17.7±1.1	12.3–17.7	14.8±1.5	17.2	11.7–17.8	15.1±2.1

**Table 2.** Major diagnostic characters amongst three species with a continuous or discontinuous orange line along the dorsal mid-line of the back. Data utilised for *L. micra* are from Bohlen and Šlechtov (2017).

Character	*L. brachycephala* sp.nov. (n = 22)	*L. citrauratea* (n = 29)	*L. micra* (n = 5)
Colour on the back of body	A continuous or discontinuous orange line along dorsal mid-line	A row of rounded orange spots along the dorsal mid-line	A row of rounded orange spots along the dorsal mid-line
Dorsal-fin origin	Slightly posterior to pelvic–fin insertion	Slightly anterior to or superior to pelvic–fin insertion	Slightly posterior to or superior to pelvic–fin insertion
Caudal-fin shape	Emarginate with rounded lobes; median rays 1.2–1.5 times as long as upper lobe.	Strongly forked, with broadly pointed lobes; median rays 1.7–2.3 times as long as upper lobe.	Moderately forked, with broadly pointed lobes; median rays 1.3–1.4 times as long as upper lobe.
Predorsal length	49.9–54.7	51.4–56.3	58.1–59.0
Body depth (% SL)	11.1–15.8	14.8–19.2	15.3–18.3
Head length (% SL)	18.4–22.8	22.5–26.8	23.6–25.9
Upper caudal-lobe length (% SL)	15.2–20.8	23.6–26.9	18.7–23.9
Caudal-peduncle length (% SL)	14.6–20.0	12.1–15.8	11.8–13.5
Dorsal-fin length (% SL)	9.0–11.6	13.7–18.0	11.0–14.6
Anal-fin length (% SL)	8.1–11.4	13.0–17.7	15.3–16.8

**Table 3.** Species included in this analysis with specimen voucher, sampling location and basin, haplotype and GenBank accession number; the haplotype with * means downloaded from GenBank.

Species	Specimen voucher	Sampling location	Basin	Haplotype	GenBank no.	Source
*L. brachycephala*	201909034355	Wenzhou, Zhejiang Prov.	Ou-Jiang	H1	MT747394	This study
*L. brachycephala*	201909034354	Wenzhou, Zhejiang Prov.	Ou-Jiang	H1	MT747394	This study
*L. brachycephala*	201909034353	Wenzhou, Zhejiang Prov.	Ou-Jiang	H1	MT747394	This study
*L. brachycephala*	201909034352	Wenzhou, Zhejiang Prov.	Ou-Jiang	H1	MT747394	This study
*L. brachycephala*	IHB2017056869	Quzhou, Zhejiang Prov.	Qu-Jiang	H1	MT747394	This study
*L. brachycephala*	IHB2017056870	Quzhou, Zhejiang Prov.	Qu-Jiang	H1	MT747394	This study
*L. brachycephala*	IHB2017056871	Quzhou, Zhejiang Prov.	Qu-Jiang	H1	MT747394	This study
*L. brachycephala*	IHB20181010544	Qingtian, Zhejiang Prov.	Ou-Jiang	H1	MT747394	This study
*L. brachycephala*	201909034349	Wenzhou, Zhejiang Prov.	Ou-Jiang	H2	MT747395	This study
*L. brachycephala*	IHB20181010537	Qingtian, Zhejiang Prov.	Ou-Jiang	H3	MT747396	This study
*L. tientainensis*	IHB2017056861	Jingdezhen, Jiangxi Prov.	Rao-He	H1	MT747348	This study
*L. tientainensis*	IHB2017056836	Jingdezhen, Jiangxi Prov.	Rao-He	H1	MT747348	This study
*L. tientainensis*	IHB2017056835	Jingdezhen, Jiangxi Prov.	Rao-He	H1	MT747348	This study
*L. tientainensis*	IHB2017056834	Jingdezhen, Jiangxi Prov.	Rao-He	H1	MT747348	This study
*L. tientainensis*	IHB2017056833	Jingdezhen, Jiangxi Prov.	Rao-He	H1	MT747348	This study
*L. tientainensis*	201909034356	Linhai, Zhejiang Prov.	Ling-Jiang	H1	MT747348	This study
*L. tchangi*	IHB2018099882	Shaoxing, Zhejiang Prov.	Cao'e-Jiang	H1	MT747349	This study
*L. tchangi*	IHB2018099861	Shaoxing, Zhejiang Prov.	Cao'e-Jiang	H1	MT747349	This study
*L. tchangi*	201909034361	Shaoxing, Zhejiang Prov.	Cao'e-Jiang	H1	MT747349	This study
*L. tchangi*	IHB2018099848	Quzhou, Zhejiang Prov.	Qu-Jiang	H2	MT747350	This study
*L. tchangi*	IHB201904029046	Hangzhou, Zhejiang Prov.	Qiantang-Jiang	H2	MT747350	This study
*L. tchangi*	IHB201904029045	Hangzhou, Zhejiang Prov.	Qiantang-Jiang	H2	MT747350	This study
*L. tchangi*	201904028852	Hangzhou, Zhejiang Prov.	Qiantang-Jiang	H2	MT747350	This study
*L. tchangi*	IHB2018099847	Quzhou, Zhejiang Prov.	Qu-Jiang	H3	MT747351	This study
*L. tchangi*	IHB2018099846	Quzhou, Zhejiang Prov.	Qu-Jiang	H4	MT747352	This study
*L. tchangi*	IHB2018099845	Quzhou, Zhejiang Prov.	Qu-Jiang	H4	MT747352	This study
*L. tchangi*	IHB2018099844	Quzhou, Zhejiang Prov.	Qu-Jiang	H5	MT747353	This study
*L. tchangi*	IHB201904029047	Hangzhou, Zhejiang Prov.	Qiantang-Jiang	H6	MT747354	This study
*L. tchangi*	IHB201904029044	Hangzhou, Zhejiang Prov.	Qiantang-Jiang	H6	MT747354	This study
*L. taeniops*	IHB2017056865	Nanchang, Jiangxi Prov.	Gan-Jiang	H1	MT747355	This study
*L. taeniops*	IHB2017056864	Nanchang, Jiangxi Prov.	Gan-Jiang	H2	MT747356	This study
*L. taeniops*	201711015714	Yiyang, Hunan Prov.	Zi-Shui	H2	MT747356	This study
*L. taeniops*	201711015711	Yiyang, Hunan Prov.	Zi-Shui	H2	MT747356	This study
*L. taeniops*	201711015710	Yiyang, Hunan Prov.	Zi-Shui	H2	MT747356	This study
*L. taeniops*	2017101867	Yuanjiang, Hunan Prov.	Zi-Shui	H2	MT747356	This study
*L. taeniops*	2017101866	Yuanjiang, Hunan Prov.	Zi-Shui	H2	MT747356	This study
*L. taeniops*	201711010290	Yiyang, Hunan Prov.	Zi-Shui	H2	MT747356	This study
*L. taeniops*	201711010026	Yiyang, Hunan Prov.	Zi-Shui	H2	MT747356	This study
*L. taeniops*	IHB2017056863	Nanchang, Jiangxi Prov.	Gan-Jiang	H3	MT747357	This study
*L. taeniops*	201711015671	Yiyang, Hunan Prov.	Zi-Shui	H3	MT747357	This study
*L. taeniops*	201801016175	Yiyang, Hunan Prov.	Zi-Shui	H4	MT747358	This study
*L. taeniops*	2017101865	Yuanjiang, Hunan Prov.	Zi-Shui	H5	MT747359	This study
*L. rubrilabris*	201904028870	Neijiang, Sichuan Prov.	Upper Chang-Jiang	H1	MT747360	This study
*L. rubrilabris*	201904028867	Neijiang, Sichuan Prov.	Upper Chang-Jiang	H2	MT747361	This study
*L. rubrilabris*	201904028858	Neijiang, Sichuan Prov.	Upper Chang-Jiang	H2	MT747361	This study
*L. rubrilabris*	201904028866	Neijiang, Sichuan Prov.	Upper Chang-Jiang	H3	MT747362	This study
*L. rubrilabris*	201904028865	Neijiang, Sichuan Prov.	Upper Chang-Jiang	H4	MT747363	This study
*L. rubrilabris*	201904028863	Neijiang, Sichuan Prov.	Upper Chang-Jiang	H5	MT747364	This study
*L. punctata*	201909037450	Baise, Guangxi Prov.	Zhu-Jiang	H1	MT747365	This study
*L. punctata*	018099887	Liuzhou, Guangxi Prov.	Zhu-Jiang	H1	MT747365	This study
*L. punctata*	201909037448	Baise, Guangxi Prov.	Zhu-Jiang	H2	MT747366	This study
*L. punctata*	201909037447	Baise, Guangxi Prov.	Zhu-Jiang	H2	MT747366	This study
*L. punctata*	201909037446	Baise, Guangxi Prov.	Zhu-Jiang	H3	MT747367	This study
*L. punctata*	201909037445	Baise, Guangxi Prov.	Zhu-Jiang	H4	MT747368	This study
*L. punctata*	018099886	Liuzhou, Guangxi Prov.	Zhu-Jiang	H4	MT747368	This study
*L. punctata*	018099885	Liuzhou, Guangxi Prov.	Zhu-Jiang	H5	MT747369	This study
*L. pellegrini*	IHB2018099886	Liuzhou, Guangxi Prov.	Zhu-Jiang	H1	MT747370	This study
*L. pellegrini*	IHB2018099885	Liuzhou, Guangxi Prov.	Zhu-Jiang	H2	MT747371	This study
*L. pellegrini*	IHB2018099884	Liuzhou, Guangxi Prov.	Zhu-Jiang	H2	MT747371	This study
*L. pellegrini*	IHB2018099840	Liuzhou, Guangxi Prov.	Zhu-Jiang	H2	MT747371	This study
*L. pellegrini*	IHB2018099839	Liuzhou, Guangxi Prov.	Zhu-Jiang	H2	MT747371	This study
*L. pellegrini*	201909034360	Liuzhou, Guangxi Prov.	Zhu-Jiang	H2	MT747371	This study
*L. pellegrini*	201909034359	Liuzhou, Guangxi Prov.	Zhu-Jiang	H2	MT747371	This study
*L. pellegrini*	201909034358	Liuzhou, Guangxi Prov.	Zhu-Jiang	H2	MT747371	This study
*L. microphthalma*	201904028856	Neijiang, Sichuan Prov.	Upper Chang-Jiang	H1	MT747372	This study
*L. microphthalma*	201904028850	Neijiang, Sichuan Prov.	Upper Chang-Jiang	H1	MT747372	This study
*L. microphthalma*	201904028855	Neijiang, Sichuan Prov.	Upper Chang-Jiang	H2	MT747373	This study
*L. microphthalma*	IHB2016105308	Leshan, Sichuan Prov.	Min-Jiang	H2	MT747373	This study
*L. microphthalma*	IHB2016105306	Leshan, Sichuan Prov.	Min-Jiang	H2	MT747373	This study
*L. microphthalma*	IHB2016105311	Leshan, Sichuan Prov.	Min-Jiang	H3	MT747374	This study
*L. microphthalma*	IHB2016105307	Leshan, Sichuan Prov.	Min-Jiang	H3	MT747374	This study
*L. microphthalma*	IHB2016105310	Leshan, Sichuan Prov.	Min-Jiang	H4	MT747375	This study
*L. microphthalma*	IHB2016105309	Leshan, Sichuan Prov	Min-Jiang	H5	MT747376	This study
*L. hengyangensis*	2017042831	Hengyang, Hunan Prov	Xiang-Jiang	H1	MT747377	This study
*L. hengyangensis*	2017042828	Hengyang, Hunan Prov	Xiang-Jiang	H2	MT747378	This study
*L. guilinensis*	2015040820	Guilin, Guangxi Prov	Zhu-Jiang	H1	MT747379	This study
*L. guilinensis*	2015040812	Guilin, Guangxi Prov	Zhu-Jiang	H1	MT747379	This study
*L. guilinensis*	2015040811	Guilin, Guangxi Prov	Zhu-Jiang	H1	MT747379	This study
*L. guilinensis*	2015040810	Guilin, Guangxi Prov	Zhu-Jiang	H1	MT747379	This study
*L. guilinensis*	2015040805	Guilin, Guangxi Prov	Zhu-Jiang	H1	MT747379	This study
*L. guilinensis*	2015040818	Guilin, Guangxi Prov	Zhu-Jiang	H2	MT747380	This study
*L. guilinensis*	2015040815	Guilin, Guangxi Prov	Zhu-Jiang	H2	MT747380	This study
*L. guilinensis*	2015040813	Guilin, Guangxi Prov	Zhu-Jiang	H3	MT747381	This study
*L. guilinensis*	2015040808	Guilin, Guangxi Prov	Zhu-Jiang	H4	MT747382	This study
*L. elongata*	IHB2018059216	Leshan, Sichuan Prov	Min-Jiang	H1	MT747383	This study
*L. elongata*	IHB2018059215	Leshan, Sichuan Prov	Min-Jiang	H2	MT747384	This study
*L. elongata*	SCULE007	Unknown		H2*	NC018764	GenBank
*L. elongata*	Unknown	Luzhou, Sichuan Prov	Upper Chang-Jiang	H2*	AY625715	GenBank
*L. elongata*	IHB2018059214	Leshan, Sichuan Prov	Min-Jiang	H3	MT747385	This study
*L. elongata*	201904028849	Leshan, Sichuan Prov	Min-Jiang	H4	MT747386	This study
*L. elongata*	IAPG A214	Unknown		H5*	AY887779	GenBank
*L. elongata*	Unknown	Unknown		H6*	KY307845	GenBank
*L. citrauratea*	IHB2017056860	Nanchang, Jiangxi Prov	Gan-Jiang	H1	MT747387	This study
*L. citrauratea*	IHB2017056859	Nanchang, Jiangxi Prov	Gan-Jiang	H2	MT747388	This study
*L. citrauratea*	IHB2017056858	Nanchang, Jiangxi Prov	Gan-Jiang	H3	MT747389	This study
*L. citrauratea*	201711016295	Nanxian, Hunan Prov	Donngting Lake	H3	MT747389	This study
*L. citrauratea*	201711015674	Nanxian, Hunan Prov	Donngting Lake	H3	MT747389	This study
*L. citrauratea*	IHB2017056857	Nanchang, Jiangxi Prov	Gan-Jiang	H4	MT747390	This study
*L. citrauratea*	201711016297	Nanxian, Hunan Prov	Donngting Lake	H5	MT747391	This study
*L. citrauratea*	201711016296	Nanxian, Hunan Prov	Donngting Lake	H6	MT747392	This study
*L. citrauratea*	201711015675	Nanxian, Hunan Prov	Donngting Lake	H6	MT747392	This study
*L. citrauratea*	201711015716	Nanxian, Hunan Prov	Donngting Lake	H7	MT747393	This study
*L. posterodorsalis*	Unknown	Xiaoxi, Hunan Prov	Yuan-Jiang	H1*	MH922928	GenBank
*P. fasciata*	Unknown	Unknown		H1*	AY625710	GenBank
*P. lijiangensis*	Unknown	Chenxi, Hunan Prov	Yuan-Jiang	H1*	AY625713	GenBank

Genomic DNA was extracted from fin clips stored in ethanol using the TIANamp Genomic DNA Kit (Tiangen Biotech, Beijing) with the recommended protocol. The cyt b gene was amplified by primers L14724 (GACTTGAAAAACCACCGTTG) and H15915 (CTCCGATCTCCGGATTACAAGAC) adopted from Xiao et al. (2001), with 1 μl of each primer, 1 μl template DNA, 12.5 μl Master mix Taq (Beijing TsingKe Biotech Co. Ltd.) and 9.5 μl double distilled water (dd H_2_O) for a total reaction volume of 25 µl. The thermocycling conditions were as follows: initial denaturation for 4 min at 94 °C, denaturation for 50 s at 94 °C, annealing for 50 s at 55 °C and extension for 1 min at 72 °C. After 34–35 cycles, the final extension was done at 72 °C for 10 min and the PCR product was preserved at 4 °C. Sequencing was carried out by the Tianyihuiyuan Biotechnology Company.

A total of 98 cyt b sequences were generated from 12 species of *Leptobotia*. These sequences were used for phylogenetic analysis together with five sequences from two congeneric species (*L. posterodorsalis* Lan & Chen, 1992 and *L. elongata*) and two sequences serving as outgroup (*Parabotia fasciata* Dabry de Thiersant, 1872 and *P. lijiangensis* Chen, 1980) downloaded from GenBank (Table 3).

The sequences were aligned utilising MAFFT version 7 (Katoh and Standley 2013) and ends trimmed, for a total alignment length of 1060 bp. The genetic distance, based on the uncorrected p-distance model (Kumar et al. 2016), was calculated with MEGA 7.0. DNASP v.5 was utilised to filter the haplotype (Librado and Rozas 2009).

PhyloSuite (Zhang et al. 2020) was used for phylogenetic analyses. The selection of the best-fit model of nucleotide evolution based on Akaike’s Information Criterion was performed in ModelFinder (Kalyaanamoorthy et al. 2017). MrBayes 3.2.6 (Ronquist et al. 2012) was utilised for Bayesian analysis with the selected model: GTR+I+G+F, applying the optimal nucleotide evolution model and the MCMC method with four chains (three hot chains and one cold chain) running simultaneously for 6,000,000 generations to calculate posterior probability. Trees were sampled for every 1000 cycles. The initial 25% of sampled data were discarded as burn-in. Sufficient mixing of the chains was regarded to be reached when the average standard deviation of split frequencies was below 0.01.

## Taxonomy

### Leptobotia citrauratea

Taxon classificationAnimaliaCypriniformesBotiidae

Nichols, 1925

400CFE19-2817-5B5C-9626-216CC7209DA3

(Fig. 1a–c)

Botia citrauratea Nichols, 1925: 5 [Tungting [now Dongting] Lake, Hunan Province
Leptobotia elongata : Chen, 1980: 14 (no localities). Kottelat, 2004:15 (no localities); 2012:16 (no locality)
Leptobotia citrauratea : Nalbant, 2002: 316 (no localities). Bohlen & Šlechtová, 2017: 90 (Nanchang City, Jiangxi Province)

#### Material examined.

*Leptobotia citrauratea*: AMNH 8402, holotype, 50 mm SL; China: Hunan Province: Dungting Lake (photograph examined); collected by Clifford H. Pope, 29 December 1921; IHB 2017100260-65, 201801026314, 201711016295, 201711015676, 201711015673-74, 201711015715-16, 201711016204, 201707028880, 201711015718, 201707028888, topotypes, 17 specimens, 47.0–65.3 mm SL; China: Hunan Province: Nanxian County: Dongting Lake; 29°2'29"N, 112°18'22"E; collected by C.T An, November 2017; IHB 2017056850-60, 2017056862, 12 specimens, 33.3–42.7 mm SL; China: Jiangxi Province: Nanchang City: Gan-Jiang, an effluent of Poyang Lake; 28°32'12"N, 115°49'24"E; collected by D.M. Guo, November 2019.

**Figure 1.**
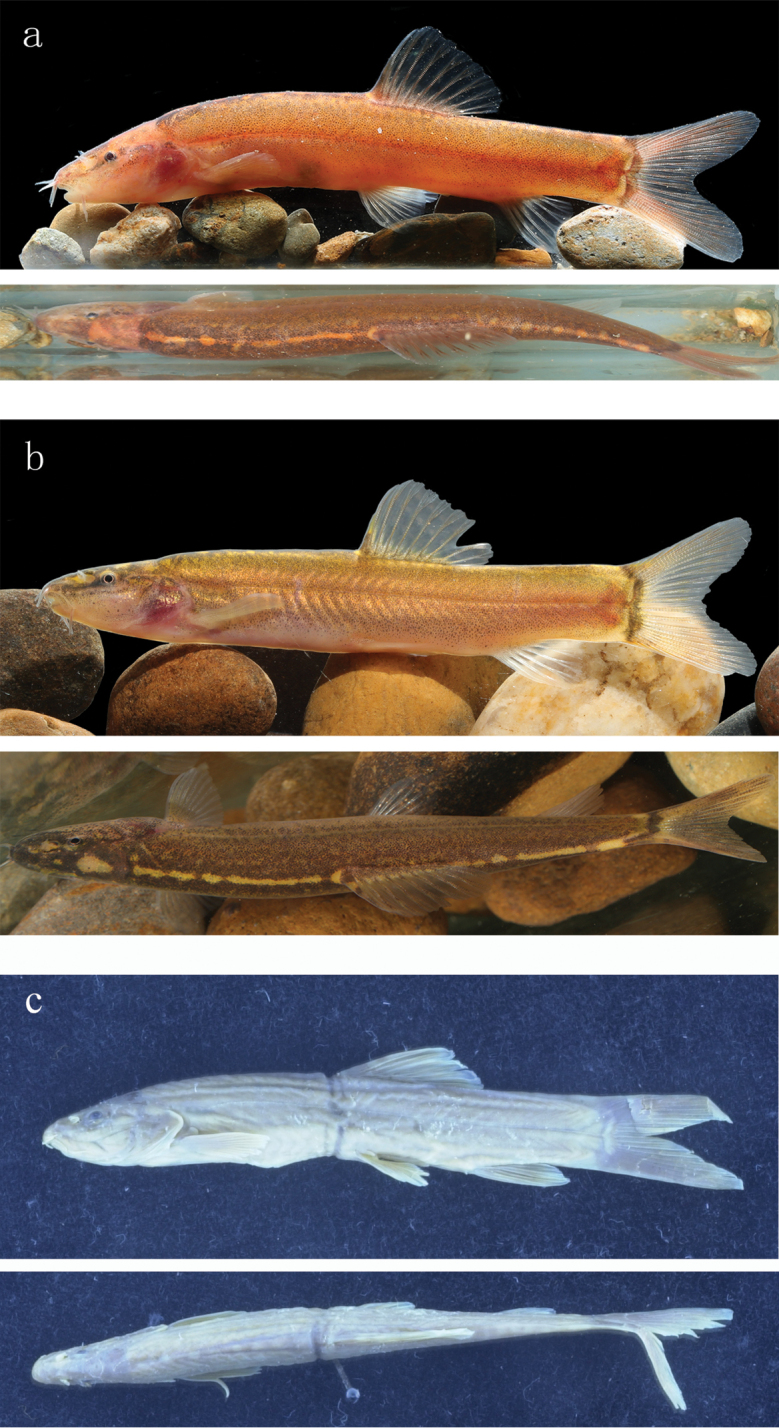
Lateral (upper) and dorsal (lower) view of *L. citrauratea* for freshly-caught specimens **a**IHB 2017100265, topotype, 53.0 mm SL, China, Hunan Province, Yiyang City, Nanxian County, Dongting Lake **b**IHB 2017056850, 38.1 mm SL, China, Jiangxi Province, Nanchang City, Gan-Jiang **c** AMNH 8402, holotype, 50 mm SL, China, Hunan Province, Dungting (presently Dongting) Lake (photos by Department of Ichthyology, American Museum of Natural History).

#### Diagnosis.

**Figure 2.**
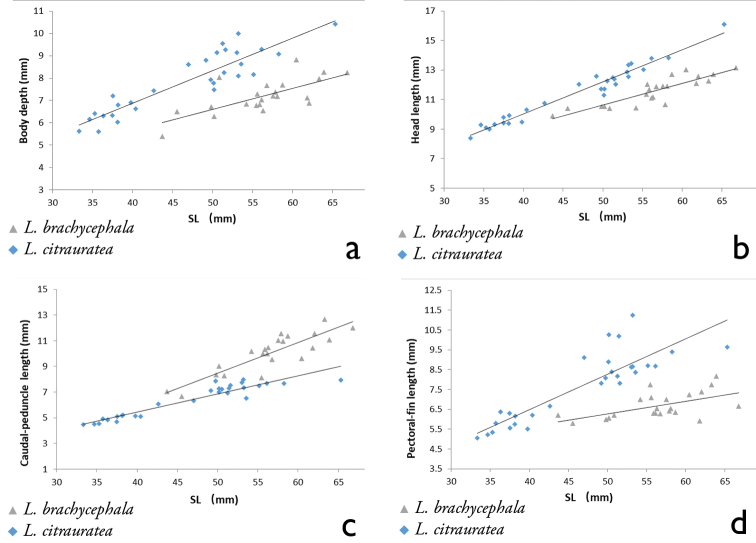
Relationship between body depth and SL (**a**), between head length and SL (**b**), between caudal-peduncle length and SL (**c**) and between pectoral-fin length and SL (**d**) for two closely-related species: *L. brachycephala* (gray triangle) and *L. citrauratea* (light blue diamond).

*Leptobotia citrauratea* shares with *L. micra* and *L. brachycephala* the unique presence of a row of orange spots or an orange stripe along the dorsal mid-line of the body, extending from the nape to the caudal-fin base. It differs from *L. micra* and *L. brachycephala* by having a deeply forked (vs. emarginate) caudal fin (length of median rays 1.7–2.3 times in length of upper lobe vs. 1.3–1.4 in *L. micra* and 1.2–1.5 in *L. brachycephala*), pelvic fin inserted slightly posterior or inferior (vs. slightly anterior in *L. brachycephala*) to the dorsal-fin origin, a longer head (22.5–26.8% SL vs. 18.4–22.8% SL in *L. brachycephala*) and a shorter predorsal distance (51.4–56.3% SL vs. 58.1–59% SL in *L. micra*) (Table 2, Fig. 2).

#### Description.

Morphometric data for specimens examined in Tables 1, 2. See Fig. 1 for lateral and dorsal view of body. Body slender, strongly compressed laterally, with maximum depth at dorsal-fin origin. Predorsal body profile slightly convex. Ventral profile of head slightly concave or straight; ventral profile of body almost straight or slightly concave from pectoral-fin insertion to anal-fin origin and slightly convex from anal-fin origin to caudal-fin base. Lateral line nearly complete, extending along mid-lateral of body. Cheek and trunk covered with minute scales.

***Head*** short, compressed laterally, length greater than maximum body depth. Snout slightly concave in lateral view, slightly shorter than postorbital head. Eye small, dorsolateral, in upper half of head; diameter less than interorbital space. Mouth inferior, with opening laterally extended to vertical through anterior margin of nostril. Button-like fleshy protrusion in gular region absent. Two rostral barbels at tip of snout. Maxillary barbel in corner of mouth, reaching beyond vertical through posterior margin of nostrils, not or just approaching to level of anterior margin of eye. Simple suborbital spine ventral to anterior margin of eye, reaching posterior margin of eye.

***Fin rays*** flexible. Dorsal fin with 4 unbranched and 8 branched rays; distal margin slightly concave; origin slightly anterior to or superior to pelvic-fin insertion and closer to caudal-fin base than to snout tip. Pectoral fin with 1 unbranched and 10–11 branched rays, tip of depressed fin extending about midway between pectoral-fin and pelvic-fin insertion. Pelvic fin with 1 unbranched and 7 branched rays, reaching about half of distance between pelvic-fin insertion and anal-fin origin and just reaching anus. Anus closer to anal-fin insertion than pelvic-fin insertion. Anal fin with 3 unbranched and 5 branched rays, tip of depressed fin not extending to caudal-fin base; distal margin slightly concave. Caudal fin strongly forked, median fin rays 1.7–2.3 times as long as lobes; upper and lower lobes broadly pointed and almost equal in length and shape.

#### Colouration.

In freshly-collected specimens, ground colour of head and body yellowish-brown or orange; lateral head and flank faintly peppered with dark grey flecks. Dorsal side of head and body dark with some rounded light orange spots usually fused to form an orange stripe extending along mid-line of dorsum from nape to caudal-fin base. Anterior to orange spots or light stripe, an orangish stripe present between eye and nape. Faint dark grey stripe extending from snout tip to anterior margin of eye. Grey bar, similar in width to eye diameter, present on caudal-fin base. In some specimens, caudal fin hyaline, in others with dark grey stripes. Single row of faint dark grey stripes present in dorsal fin.

In specimens preserved in formalin, ground colour slightly faded, not presenting vivid yellowish-brown or orange, but becoming whitish-grey and peppered with dark flecks. Dorsum and head darkened. Orange spots along mid-line of dorsum white. Dorsal, pectoral, pelvic and anal fins greyish-yellow at base with white distal margins. Caudal fin dusky.

#### Geographical distribution and habitat.

**Figure 3.**
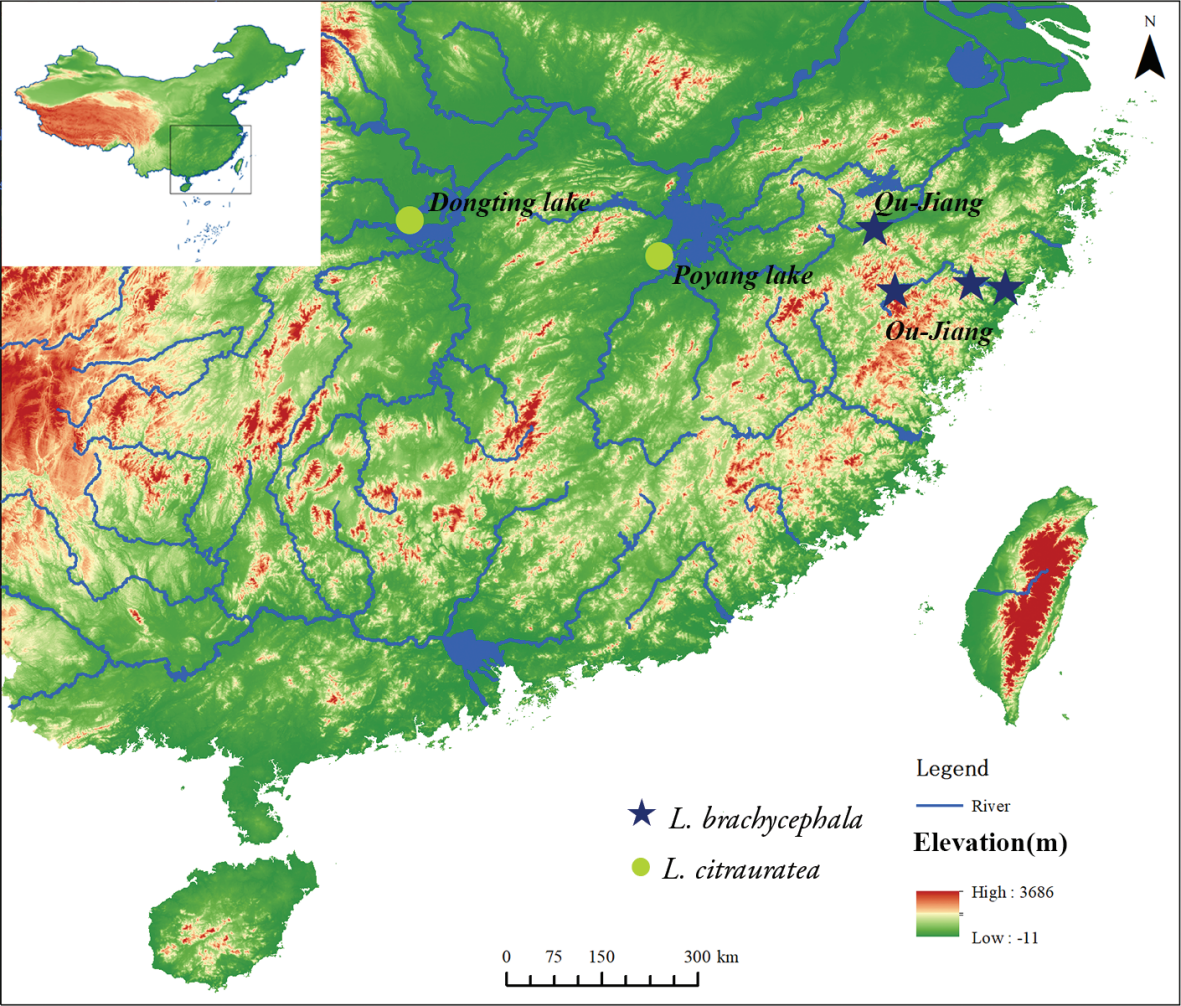
Collection localities of *L. brachycephala* (dark blue star) and *L. citrauratea* (light green dot).

*Leptobotia citrauratea* is known from Dongting Lake in Hunan Province and the Gan-Jiang, discharging into Poyang Lake, in Jiangxi Province, southern China (Fig. 3). The specimens here described were collected in deep and slow-running water with mixed substrate. Syntopic fish species included *Saurogobio dabryi* Bleeker, 1871, *Parabotia banarescui* (Nalbant, 1965) and *Hemibarbus maculatus* Bleeker, 1871.

### Leptobotia brachycephala sp. nov.

Taxon classificationAnimaliaCypriniformesBotiidae

55DB1420-E1E5-50D3-B8D1-DCCE5CEC1983

http://zoobank.org/4A74D0D5-5A3B-4458-8227-B67A591A4143

(Fig. 4a, b)

#### Holotype.

IHB 201909037510, 63.9 mm SL; China: Zhejiang Province: Qingtian County: a stream discharging into the Ou-Jiang; 28°10'20"N, 120°12'51"E; collected by E Zhang and D.M. Guo, 8 October 2018.

#### Paratypes.

IHB 2017056858, 2017056869-80, 13 specimens, 54.2–66.8 mm SL; China: Zhejiang Province: Quzhou City: a stream flowing into the Qu-Jiang; 28°57'6"N, 118°51'15"E; collected by D.M. Guo, 15 December 2019.

#### Non-types examined.

IHB 64VI410-15, 930138-39, 8 specimens, 43.7–60.5 mm SL; China: Zhejiang Province: Longquan City: a stream flowing into the Ou-Jiang; 28°4'12"N, 119°6'54"E; collected in 1964 and 1983.

#### Diagnosis.

**Figure 4.**
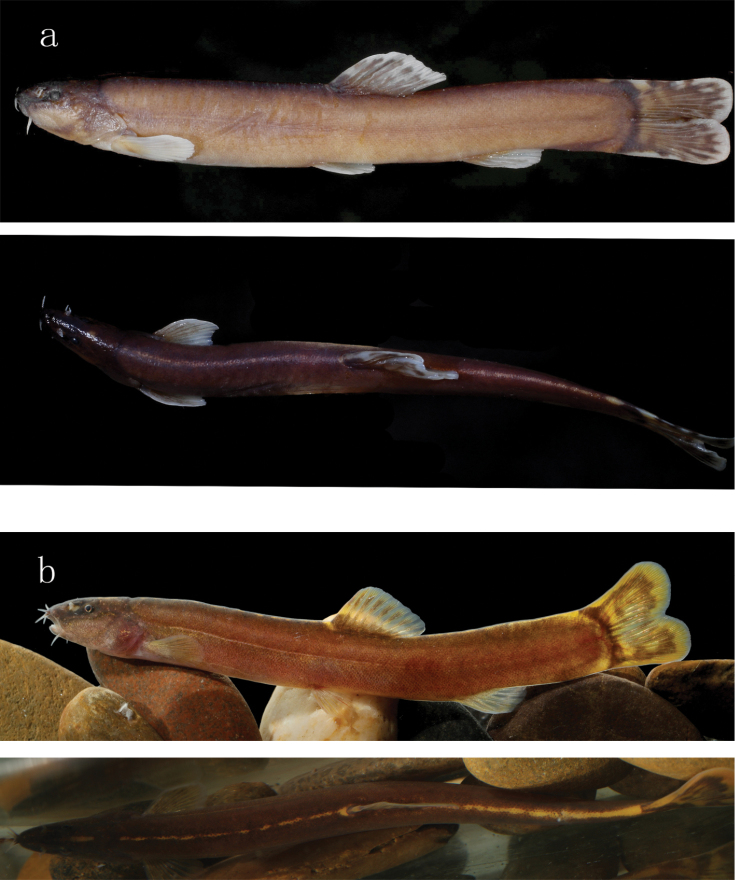
Lateral (upper) and dorsal (lower) view of body for *L. brachycephala*: **a**IHB 201909037510, holotype, 63.9 mm SL, China, Zhejiang Province, Lishui City, Qingtian County, Ou-Jiang, kept in formalin preservative after capture **b**IHB 2017056867, paratype, 62.5 mm SL, freshly collected from Qu-Jiang at Kecheng District, Quzhou City, Zhejiang Province.

*Leptobotia brachycephala*, together with *L. citrauratea* and *L. micra*, is distinguished from all other congeneric species by the presence (vs. absence) of a row of orange dots or an orange stripe extending along the dorsal mid-line of the body from the nape to the caudal-fin base (Fig. 4a, b: lower). It differs from *L. citrauratea* and *L. micra* in having an emarginate (vs. forked) caudal fin with two rounded (vs. broadly pointed) lobes (Figs 1a–c, 4a, b: upper), a shorter head (18.4–22.8% SL vs. 22.5–26.8% SL in *L. citrauratea* and 23.6–25.9% SL in *L. micra*), a slender caudal peduncle (14.6–20.0% SL vs. 12.1–15.8% SL in *L. citrauratea* and 11.8–13.5% SL in *L. micra*), a shorter dorsal fin (9.0–11.6% SL vs. 13.7–18% SL in *L. citrauratea* and 11.0–4.6% SL in *L. micra*) and a shorter anal fin (8.1–11.4% SL vs. 13.0–17.7% SL in *L. citrauratea* and 15.3–16.8% SL in *L. micra*) (Table 2, Fig. 2).

#### Description.

Morphometric data given in Tables 1, 2. See Fig. 4 for lateral and dorsal view of body. Body slender, strongly compressed laterally, with greatest depth at dorsal-fin origin. Dorsal profile of head rising progressively from tip of snout to nape, from there to caudal-fin base nearly straight. Ventral profile of head slightly concave; ventral profile of body almost straight or slightly concave. Lateral line nearly complete, extending along mid-lateral body to terminate in median caudal-fin rays. Cheek and trunk covered with some minute scales.

***Head*** short, compressed laterally, longer than maximum body depth. Snout slightly obtuse in lateral view, slightly shorter than postorbital head. Eye small, dorsolateral, in upper half of head; diameter less than interorbital width. Mouth inferior, with opening laterally extended to vertical through anterior margin of nostril. Button-like structures in gular region absent; no median incisions in lower lip. Two rostral barbels at tip of snout. Maxillary barbel in corner of mouth, not reaching to level of anterior margin of eye. Simple suborbital spine ventral to anterior margin of eye, not or just reaching posterior margin of eye.

***Fin rays*** flexible. Dorsal fin with 4 unbranched and 8 branched rays; distal margin slightly concave; origin slightly posterior to pelvic-fin insertion and closer to caudal-fin base than to tip of snout. Pectoral fin with 1 unbranched and 10–11 branched rays, not extending to midway from pectoral-fin to pelvic-fin insertion. Pelvic fin with 1 unbranched and 7 branched rays, not extending to halfway to anal-fin origin or not reaching anus; vent closer to anal-fin origin than to pelvic-fin insertion. Anal fin with 3 unbranched and 5 branched rays, not reaching caudal-fin base; distal margin slightly concave; origin closer to pelvic-fin insertion than to caudal-fin base. Caudal fin emarginate or shallowly forked, length of median fin rays 1.3–1.5 times in length of upper lobe; caudal-fin lobes rounded; upper and lower ones almost equal in length and shape.

#### Colouration.

In freshly-caught specimens, ground colour of head and body brownish-yellow; darker in upper half of head, but lighter in lower half of head and ventral side of body. A continuous or discontinuous orange stripe along mid-line of dorsum from nape to caudal-fin base, becoming more conspicuous towards caudal-fin base. Anterior to orange stripe, a short orange stripe present between eye and anterior margin of nape. A dark grey stripe on basal portion of dorsal fin and one stripe on dorsal fin. A dark grey band at caudal-fin base. Some irregular black stripes on caudal fin with hyaline distal edge. Distinct stripes absent from other fins. Specimens stored in formalin with ground colour of head and body pale brown. Discontinuous or continuous white line along dorsal mid-line of body also faded.

#### Geographical distribution and habitat.

**Figure 5.**
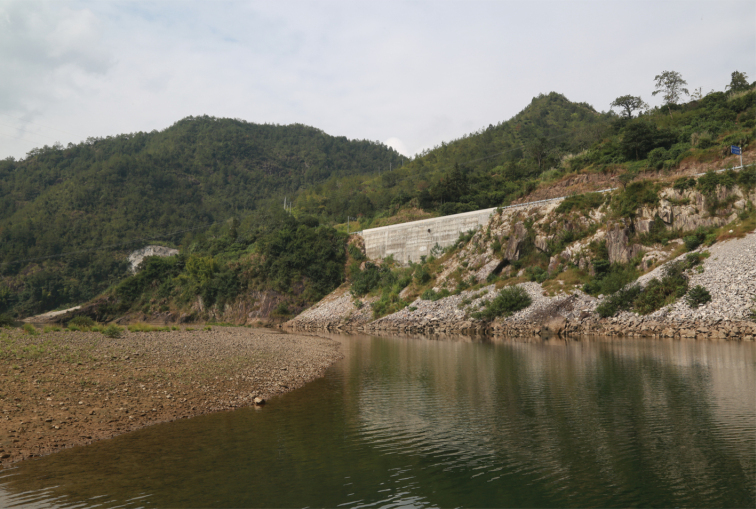
Type locality of *L. brachycephala*: fast-flowing clear water with mixed substrate including pebbles, gravels and boulders.

*Leptobotia brachycephala* is known only from the Ou-Jiang and Qu-Jiang, two coastal rivers of southern Zhejiang Province, China (Fig. 3). Type specimens were caught in fast-flowing clear water with mixed substrate including pebbles, gravels and boulders (Fig. 5). Syntopic species included *Sarcocheilichthys parvus* Nichols, 1930, *Acrossocheilus wenchowensis* Wang, 1935, *Cobitis sinensis* Sauvage & Dabry de Thiersant, 1874 and *Rhinogobius giurinus* (Rutter, 1897).

#### Explanation of name.

The specific epithet is a Latin version of the Greek words βραχύς (short) and κεφαλά (head), with reference to the short head; to be treated as a noun in apposition.

#### Genetic comparisons.

A total of 50 unique haplotypes were detected amongst the 103 cyt b sequences of species of *Leptobotia* (Table 3). The fragment contained 784 conserved sites, 276 variable sites, 233 parsimony informative sites and 43 singleton sites. The average frequency of four nucleotides of *L. citrauratea* was A = 27.8%, T = 27.8%, C = 30.3% and G = 14.1%. The intraspecific genetic distance, calculated for sampled species of *Leptobotia* with more than one haplotype, varied from 0.1% to 0.8%. *Leptobotia citrauratea* is separated from other congeneric species by high genetic divergences of 2.9% to 10.5%; its intraspecific genetic distance was 0.4%. The genetic distance of *L. brachycephala* versus congeneric species ranged from 2.9% to 10.6%; its intraspecific genetic distance was 0.1% (Table 4).

**Table 4.** Genetic distances of the cyt b gene computed by MEGA 7 amongst 13 analysed species of *Leptobotia*.

Species	Intraspecific	1	2	3	4	5	6	7	8	9	10	11	12
1 *L. tientainensis*	n/c												
2 *L. tchangi*	0.003	0.078											
3 *L. taeniops*	0.003	0.081	0.058										
4 *L. rubrilabris*	0.002	0.077	0.057	0.055									
5 *L. punctata*	0.004	0.106	0.104	0.111	0.096								
6 *L. pellegrini*	0.002	0.077	0.064	0.073	0.061	0.107							
7 *L. microphthalma*	0.004	0.067	0.069	0.067	0.061	0.092	0.077						
8 *L. hengyangensis*	0.008	0.069	0.041	0.057	0.047	0.098	0.059	0.059					
9 *L. guilinensis*	0.002	0.018	0.086	0.086	0.080	0.110	0.080	0.069	0.079				
10 *L. elongata*	0.002	0.073	0.071	0.073	0.066	0.096	0.067	0.053	0.067	0.073			
11 *L. citrauratea*	0.004	0.079	0.075	0.069	0.062	0.105	0.076	0.061	0.069	0.080	0.071		
12 *L. posterodorsalis*	n/c	0.066	0.069	0.074	0.068	0.104	0.069	0.070	0.066	0.069	0.068	0.065	
13 *L. brachycephala*	0.001	0.076	0.072	0.071	0.064	0.106	0.065	0.064	0.066	0.078	0.067	0.029	0.060

**Figure 6.**
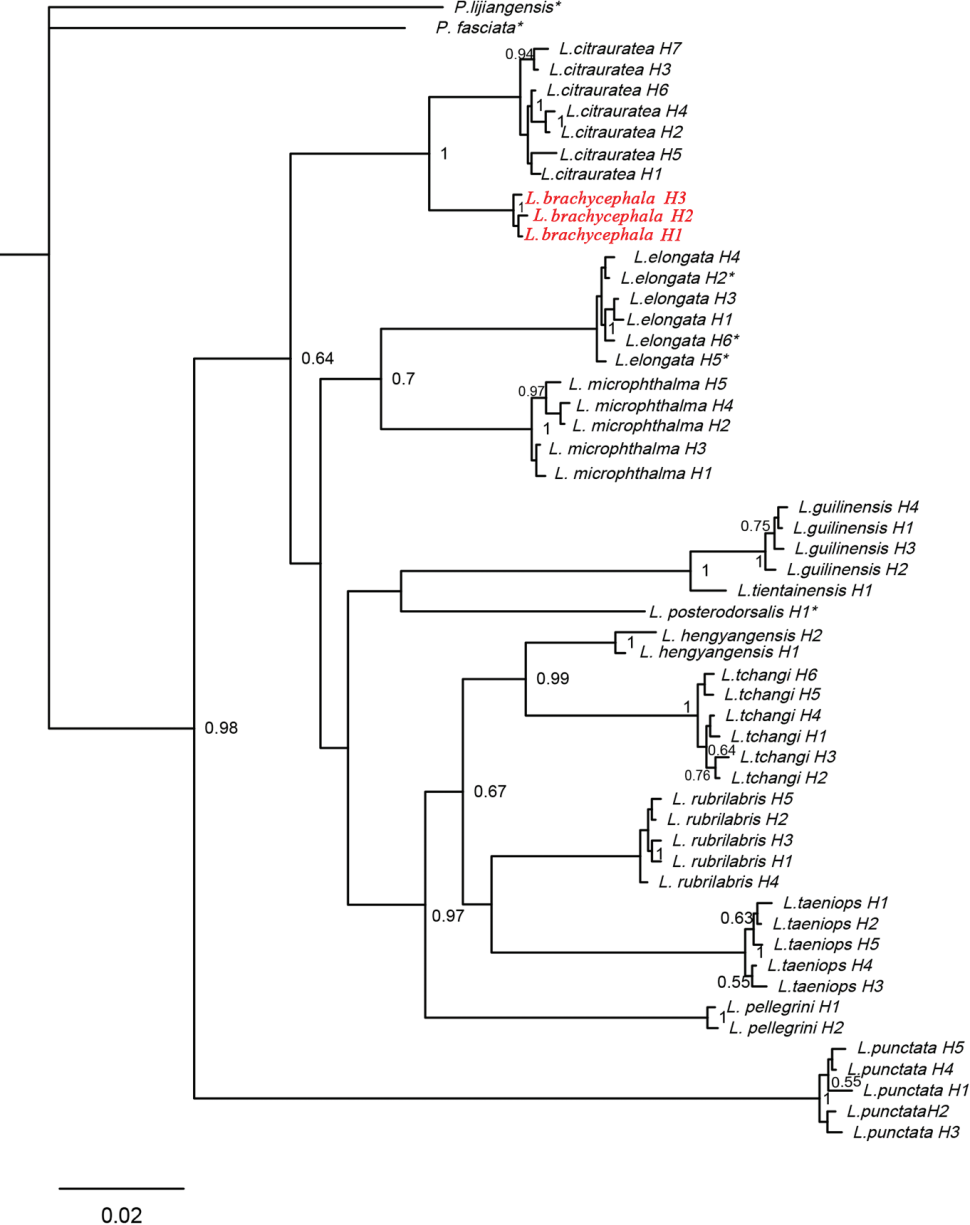
Bayesian Inference tree inferred from the cyt b gene for 13 analysed species of *Leptobotia*. Nodal numbers are posterior probability values greater than 50%

In the Bayesian 50% majority consensus tree, samples of *L. brachycephala* formed a well-supported (100% pp) lineage and so did those of both *L. citrauratea* and *L. elongata*. *L. citrauratea* was distantly allied to *L. elongata*, but robustly supported by 100% pp to be sister to *L. brachycephala* (Fig. 6).

#### Comparative morphometrics.

**Figure 7.**
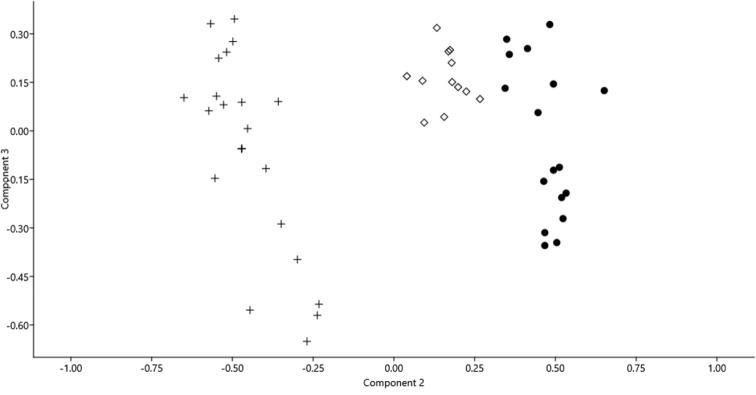
Scatter plot of the principal components II and principal components III, extracted from morphometric data of *L. brachycephala*: Ou-Jiang (plus symbol). *L. citrauratea*: Dongting Lake (black circle) and Gan-Jiang (white diamond).

In the Principal Component Analysis of specimens of *L. citrauratea* from Dongting Lake and the Gan-Jiang and *L. brachycephala* from the Ou-Jiang and Qu-Jiang, the first three components explained 91.60% of the total variance, of which 64.58%, 19.61% and 7.41% were explained, respectively by PC 1, PC 2 and PC 3 (Table 5). In the scatterplot of PC 2 and PC 3 loadings (Fig. 7), specimens of *L. citrauratea* and *L. brachycephala* constituted two distinct clusters separated on the PC 2 axis. Six characters with main loading on this axis were caudal-peduncle length, anal-fin length, dorsal-fin length, upper caudal-lobe length, pectoral-fin length and vent to anal-fin distance. Except for the last character, all of them exhibited differences in the morphometric comparisons. Table 2 and Fig. 2 show the main morphological characters.

**Table 5.** Loadings of morphological traits on the ﬁrst three principal components. Variables in bold indicate higher loading values.

Variable	PC1	PC2	PC3
Standard length	0.224	-0.150	-0.079
Body depth	0.164	0.155	0.060
Body width at dorsal origin	0.163	0.123	**0.287**
Head length	0.169	0.050	-0.002
Head depth at nape	0.165	0.173	0.108
Head depth at eye	0.148	0.150	0.112
Caudal-peduncle length	0.288	-**0.445**	-0.100
Caudal-peduncle depth	0.206	-0.138	-0.026
Caudal-peduncle width	0.169	-0.237	**0.862**
Dorsal-fin length	0.197	**0.375**	-0.025
Pectoral-fin length	0.187	**0.241**	-0.118
Pelvic-fin length	0.176	0.144	-0.124
Anal-fin length	0.199	**0.381**	0.014
Upper caudal-lobe length	0.162	**0.259**	-0.031
Predorsal length	0.218	-0.122	-0.082
Prepectoral length	0.181	0.029	-0.025
Prepelvic length	0.228	-0.039	-0.030
Preanal length	0.220	-0.075	-0.052
Vent to anal-fin origin	0.325	-**0.242**	-0.216
Pelvic-fin insertion to anal-fin origin	0.256	-0.130	-0.059
Snout length	0.186	0.076	0.072
Postorbital head length	0.170	-0.035	-0.087
Eye diameter	0.180	0.035	-0.023
Interorbital width	0.156	0.209	0.099
Median caudal-ray length	0.147	-0.168	-0.119
**Cumulative variance (%)**	64.6	19.6	7.4

## Discussion

Nichols et al. (1925) described the colour of *L. citrauratea* as “purplish brown; yellowish below”. This colouration is shared with freshly-caught specimens of this species from the Dongting Lake (type locality) and Poyang Lake systems (Fig. 1). Our examination of these topotypical specimens and a photograph of the holotype (AMNH 8402) (Fig. 1c) confirmed Bohlen and Šlechtová’s (2016) observation that *L. citrauratea* has some round orange dots or an orange stripe along the dorsal mid-line of the body. There were no significant differences in morphometric measurements and meristic counts detected between specimens from the Dongting and Poyang Lake systems (Table 1).

**Figure 8.**
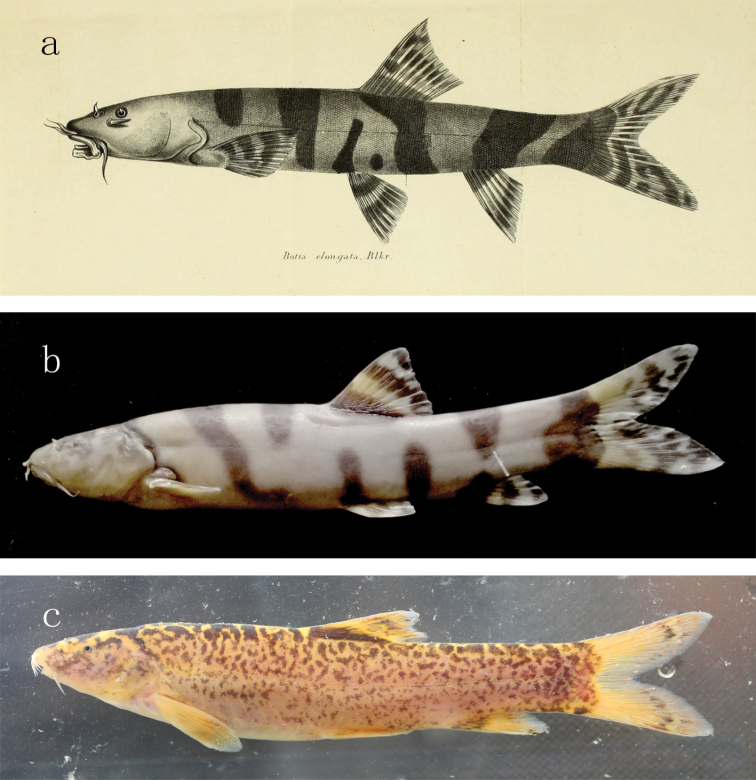
**a** a copy of Bleeker’ (1870) illustration of *L. elongata***b** lateral view of body for *L. elongata* in IHB 2018059214, 175.83 mm SL, China, Sichuan Province, Leshan City, upper Chang-Jiang Basin, kept in formalin preservative after capture **c** lateral view of body for *L. taeniops* in IHB 2017110267, 100.2 mm SL, freshly caught from Dongting Lake at Nanxian County, Yiyang City, Hunan Province, South China.

Chen (1980) synonymised *L. citrauratea* with *L. elongata*, a species found in the mid-upper Chang-Jiang Basin, but without examination of their type specimens or even reference to topotypical specimens. This classification had been widely accepted by subsequent researchers until 2002 when Nalbant recognised *L. citrauratea* as valid. Morphological data, provided in this study, indicated that there were distinct variations between these two species. Topotypical specimens examined of *L. citrauratea* possessed a small-sized body of up to 70.0 mm SL, while the body size of *L. elongata* attained a length of 97.8 to 272.0 mm SL for available specimens caught from the upper Chang-Jiang Basin. The specimen of the species, caught by Fang (1936), reached up to 500 mm in total length. *Leptobotia citrauratea* has a series of small orange spots or an orange stripe along the dorsal mid-line of the body from the nape to the caudal-fin base, a yellowish-brown or orange ground colour of head and body and no black band crossing the dorsum (Fig. 1). This is contrast to *L. elongata* which, in light of its original account and our observation on specimens collected from the upper Chang-Jiang Basin, has a body colouration of many wide brown and transverse bands (Fig. 8a, b). Additionally, *L. citrauratea* differs from *L. elongata* in having larger eyes (diameter 9.2–12.1% HL vs. 4.2–8.4% HL; see Table 1) and pelvic fin not or just reaching (vs. exceeding) the anus. Our molecular analysis also showed that *L. citrauratea* had a 7.1% interspecific genetic distance with *L. elongata* (Table 4) and that these two species constituted two independent lineages distantly related in the phylogenetic tree, based on the cyt b gene (Fig. 6). It is thus concluded here that *L. citrauratea* is a species distinct from *L. elongata* and confined only to the mid-lower Chang-Jiang Basin (the Dongting and Poyang Lake Basins); *L. elongata* is actually an endemic species of the upper Chang-Jiang Basin.

**Figure 9.**
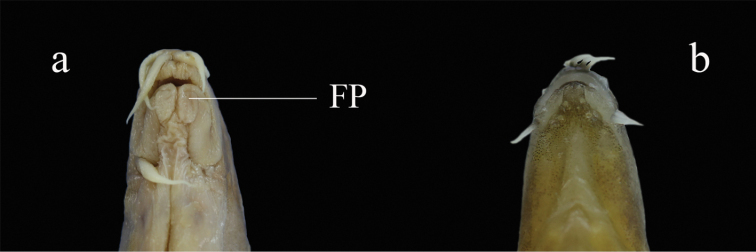
Ventral view of the mouth of two species **a***L. rubrilabris*, IHB 581-032, 150.2 mm SL, China, Chongqing City, Banan District, upper Chang-Jiang **b***L. brachycephala*, IHB 2017056872, Paratype, 66.8 mm SL, China, Zhejiang Province, Quzhou City, Kecheng District, Qu-Jiang. FP = fleshy protrusion.

Nichols et al. (1925) recognised three species of *Botia* from the Dongting Lake system: one previously-described species as *Botia rubrilabris* Dabry de Thiersant, 1872 and two new species, *B. purpurea* and *B. citrauratea*. These three species were later referred to *Leptobotia* where *B. purpurea* were synonymised with *L. taeniops* (Sauvage, 1878) (Chen 1980; Kottelat 2004, 2012). The latest report on the distribution of *L. rubirilabris* (Dabry de Thiersant, 1872) in this Lake was Anonymous (1980) who caught a single specimen of 80.0 mm SL. This specimen, in light of their description, has a button-like fleshy protrusion in the gular area, a character diagnostic for *L. rubirilabris* within this genus (Chen 1980); thus, it is conspecific with this species. Recent field surveys, conducted by us from 2014 to 2018 in Dongting Lake, yielded no specimens of this species. Likely, it was extirpated in this Basin. *Leptobotia citrauratea* mainly differs from *L. rubrilabris*, caught from the upper Chang-Jiang Basin (its type locality), in having a shorter (vs. longer) suborbital spine just reaching (vs. far beyond) the posterior margin of the eye and no button-like fleshy protrusion in the gular area (vs. present) (Fig. 9).

The specific status of *L. brachycephala* was confirmed by its morphological and genetic distinction with closely-related congeneric species (Tables 2–4). The PCA results showed that specimens of this species from southern Zhejiang Province formed a cluster, distinct from the one formed by specimens of *L. citrauratea* from the Dongting and Poyang Lake systems (Fig. 7). Although *L. brachycephala* was robustly supported by 100% pp to be sister to *L. citrauratea*, their interspecific genetic distance was 2.9%.

The seventeen species currently included in *Leptobotia* can be subdivided into six groups, based on their body colourations. The first one is only composed of one species *L. taeniops* that has a unique body colouration of some irregular purplish-brown stripes in the shape of a worm on the flank, hence resulting in a marbled or vermiculated pattern (Fig. 8c). The second group, represented by *L. punctata* Li, Li & Chen, 2008, has a lot of irregularly-organised white spots on the flank, giving a reticulated pattern. The third group, including *L. pellegrini* Fang, 1936, *L. elongata*, *L. hengyangensis* Huang & Zhang, 1986, *L. tchangi* Fang, 1936 and *L. rubrilabris*, is characterised by having a body colouration of some broad brown-black blotches on the body or saddles on the dorsum. The fourth group is formed by the following four species: *L. posterodorsalis*, *L. bellacauda* Bohlen & Šlechtová, 2016, *L. tientainensis* (Wu, 1930) and *L. microphthalma* Fu & Ye, 1983, all of them having a plain brown body and no other colour formation. The fifth group has some rounded light orange spots extending along the mid-line of the dorsum from nape to caudal-fin base and three species are included in this group: *L. micra*, *L. citrauratea* and *L. brachycephala*. The last group has narrow bands on the body or mid-line of the back and three species can be referred to this group: *L. guilinensis* Chen, 1980, *L. orientalis* and *L. flavolineata*.

## Comparative material

*Leptobotia elongata*: IHB 3609, 58199, 64V2269-70, 4 specimens, 189.6–224.7 mm SL, upper Chang-Jiang Basin at Yichang City, Hubei Province; IHB 2018059214-16, 3 specimens, 170.0–176.5 mm SL, upper Chang-Jiang Basin at Leshan City, Sichuan Province; IHB 790197, 790198-99, 3 specimens, 87.8–167.4 mm SL, upper Chang-Jiang Basin at Luzhou City, Sichuan Province; IHB 790349, 1 specimens, 161.0 mm SL, upper Chang-Jiang Basin at Yibin City, Sichuan Province; IHB 201909035604, 1 specimens, 272.0 mm SL, upper Chang-Jiang Basin at Liangshan Yi Autonomous Prefecture, Sichuan Province; IHB 585410, 501244, 73V1466, 420457, 590452-54, 580451, 8 specimens, 116.8–236.7 mm SL, upper Chang-Jiang Basin at Chongqing City; IHB 201909035958, 201909035934, 2 specimens, 100.7–110.7 mm SL, upper Chang-Jiang Basin at Zhaotong City, Yunnan Province.

*Leptobotia taeniops*: IHB 2017100254-59, 2017110267, 7 specimens, 43.6–112.9 mm SL, middle Chang-Jiang Basin at Yiyang City, Hunan Province; 201807020856, 201807028170, 2 specimens, 61.2–82.4 mm SL, middle Chang-Jiang Basin at Yueyang City, Hunan Province;

*Leptobotia rubrilabris*: IHB 581-032, 2 specimens, 150.2 mm SL, Dongting Lake at Nanxian County, Yiyang City, Hunan Province, South China;

*Leptobotia tientainensis*: IHB 74VI3347-50, 4 specimens, 70.5–91.2 mm SL, Ling-Jiang at Taizhou City, Zhejiang Province;

*Leptobotia guilinensis*: IHB 2015040803, 1 specimen, 70.3 mm SL, Zhu-Jiang at Guilin City, Guangxi Province;

*Leptobotia tchangi*: IHB 201904029044, 1 specimen, 80.4 mm SL, Qiantang-Jiang at Hangzhou City, Zhejiang Province;

*Leptobotia pellegrini*: IHB 2018099839, 1 specimen, 103.2 mm SL, Zhu-Jiang at Liuzhou City, Guangxi Province;

*Leptobotia hengyangensis*: IHB 2017042831, 2017042828, 2 specimens, 99.7–102.8 mm SL, Xiang-Jiang at Hengyang City, Hunan Province;

*Leptobotia microphthalma*: IHB 2016105306, 1 specimen, 78.3 mm SL, upper Chang-Jiang Basin at Leshan City, Sichuan Province.

Data for *L. bellacauda* and *L. micra* were taken from Bohlen and Šlechtová (2016), (2017).

## Supplementary Material

XML Treatment for Leptobotia citrauratea

XML Treatment for Leptobotia brachycephala
